# Metabolomic Profile and Functional State of Oat Plants (*Avena sativa* L.) Sown under Low-Temperature Conditions in the Cryolithozone

**DOI:** 10.3390/plants13081076

**Published:** 2024-04-11

**Authors:** Vasiliy V. Nokhsorov, Fedor F. Protopopov, Igor V. Sleptsov, Lidia V. Petrova, Klim A. Petrov

**Affiliations:** 1 Institute for Biological Problems of Cryolithozone, Siberian Branch of Russian Academy of Sciences, Division of Federal Research Centre “The Yakut Scientific Centre of the Siberian Branch of the Russian Academy of Sciences”, 41 Lenina Av., 677000 Yakutsk, Russia; neroxasg@mail.ru (I.V.S.); kap_75@bk.ru (K.A.P.); 2 Radiation Technology Laboratory, Institute of Physics and Technology, North-Eastern Federal University, 48 Kulakovskogo Str., 677000 Yakutsk, Russia; protopopov_fedor@mail.ru; 3 M.G. Safronov Yakut Scientific Research Institute of Agriculture, Division of Federal Research Centre “The Yakut Scientific Centre of the Siberian Branch of the Russian Academy of Sciences”, 23/1 Bestuzhev-Marlinskogo Str., 677000 Yakutsk, Russia; pelidia@yandex.ru

**Keywords:** oats, metabolome, leaves, low temperatures, quenching, chlorophyll fluorescence, leaf spectra

## Abstract

Oats are one of the most useful and widespread cereal crops in the world. In permafrost conditions (Central Yakutia), based on metabolic changes in late summer-sown oat plants (*Avena sativa* L.), the key processes involved in the cold acclimation of a valuable cereal species were identified. During the onset of low ambient temperatures, metabolites from leaf samples were profiled using gas chromatography with mass spectrometry (GC-MS) and were analyzed using principal component analysis (PCA). A total of 41 metabolites were identified in oat leaves. It was found that acclimation to suboptimal temperatures during the fall period leads to biochemical (accumulation of mono- and disaccharides and decrease in fatty acids and polyols) as well as physiological and biophysical changes (decrease in leaf PRI reflectance indices and chlorophyll *a* fluorescence). Therefore, the study contributes to a more holistic understanding of oat metabolism under low-temperature cryolithozone stress. It is believed that the analysis of changes in leaf reflection properties and JIP-test parameters of chlorophyll *a* fluorescence using leaf metabolomic profiling can be used in the selection of valuable varieties of cereal crops to obtain plant fodders with high nutrient contents under conditions of a sharply continental climate.

## 1. Introduction

Species of the oats genus (*Avena* L.) are some of the most important cereal crops on the globe; they are sown in about 20 million hectares of arable land [1]. One of the representatives of this genus is sown oats (*Avena sativa* L.), which is among the six cereals used for human consumption, livestock feed, forage, fodder crops, hay and silage. *A. sativa* is considered the best cereal crop due to its endurance and capacity for growth and resistance to change under unfavorable ambient factors [2]. Many of these benefits are due to the presence of multiple biologically active phytochemical substances in oat plants like phenolic compounds, flavonoids, phytosterols, carotenoids and others [3]. In addition, *A. sativa* is also responsible for the production of the rare phytochemicals that are characterized as avenanthramides, avenacosides and avenacins [4]. The most important biochemical features of cultivated plants that characterize their valuable nutritional and biomedical properties are secondary plant metabolites and the main substances of primary metabolism, such as polysaccharides, proteins and lipids.

*A. sativa* is known to be well adapted to a wide range of soil types, and for this reason it can grow better than other minor cereals on infertile soils [5]. However, oats can be sensitive to environmental temperature changes, such as hot and dry weather, low temperatures and drought [5]. In general, in the process of their development, plant organisms growing under natural conditions are permanently subjected to different unfavorable biotic, abiotic and anthropogenic factors. The impact of cold temperatures, which are the main factors determining the geographical distribution of agricultural crops on our planet, is one of the most important stress influences.

In the permafrost zone, oats are one of the leading grain-forage crops among the poor set of grain crops cultivated for fodder purposes. The climate of the cryolithozone of Yakutia is characterized by a relatively brief vegetation period (about 80–120 frost-free days per year), an unusually cold and dry climate with very low temperatures in winter (up to −60 °C) and high temperatures in summer (up to +40 °C), and the distribution of permafrost over the entire area [6]. Within a brief growing season, the plants are subjected to the impact of strong radiation from the sun, lack of water, and short-term frost on the surface of the soil in the beginning of summer and in the fall.

Studies based on model objects have led to a significant improvement in the understanding of the molecular basis of plant cold tolerance. Several hundreds of genes involved in plant response to cold stress have been identified, including genes encoding transcription factors, phosphatases and kinases [7,8,9,10]. As a rule, in natural conditions, temperature decrease is gradual, allowing the plant to restructure its metabolism to form resistance to hypothermia [11]. However, a comparative analysis of *Lotus* plants revealed that only a small fraction of abiotic stress-induced metabolites were shared between model and cultivated species, indicating a complex transfer of information from model plants to crops [12]. This emphasizes the importance of studying physiological and biochemical changes in crops under natural growing conditions when exposed to low quenching ambient temperatures.

In this regard, late-sown *A. sativa* is a convenient model for revealing adaptive responses of metabolic processes to the decrease in air temperature in the fall period, which occurs in natural conditions in the cryolithozone, and we believe that this study will be a good theoretical basis for obtaining and producing succulent feeds that may be applied in the rations of many livestock in the northern regions.

Some of the informative parameters characterizing the specific response of a plant organism to temperature decrease are adaptive changes in the primary and secondary metabolites of plants. However, adaptive changes in metabolites in herbaceous plants and their role in the regulation of vital activity during the period of autumn cold hardening with low positive and especially the first negative temperatures in the environment in cryolithozone conditions remain unstudied.

In this regard, the aim of this study was to investigate the metabolomic profile, optical reflection properties and functional state of spring oat leaves at the onset of low positive and negative (suboptimal) temperatures in the autumn period in the cryolithozone area.

## 2. Results

### 2.1. Periods of Phenological Phases of Development of Late Summer-Sown A. sativa

**Figure 1:**
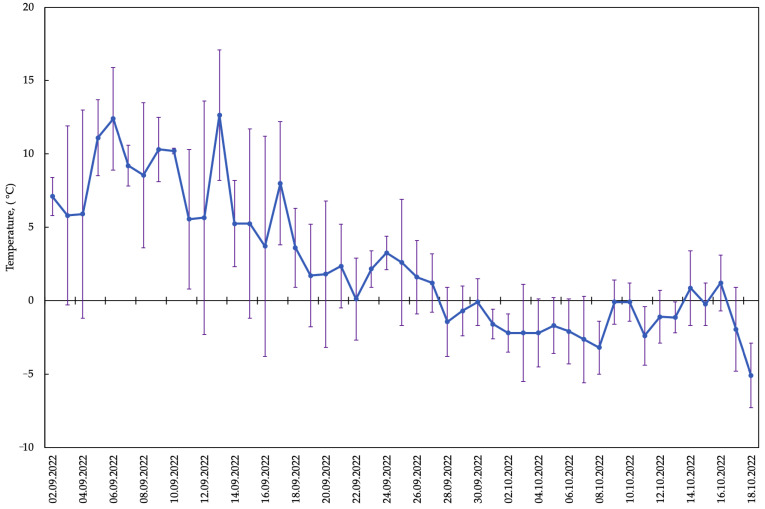
Daily mean air temperature changes during the study period (Central Yakutia, Russia). Metostation Yakutsk.

**Figure 2:**
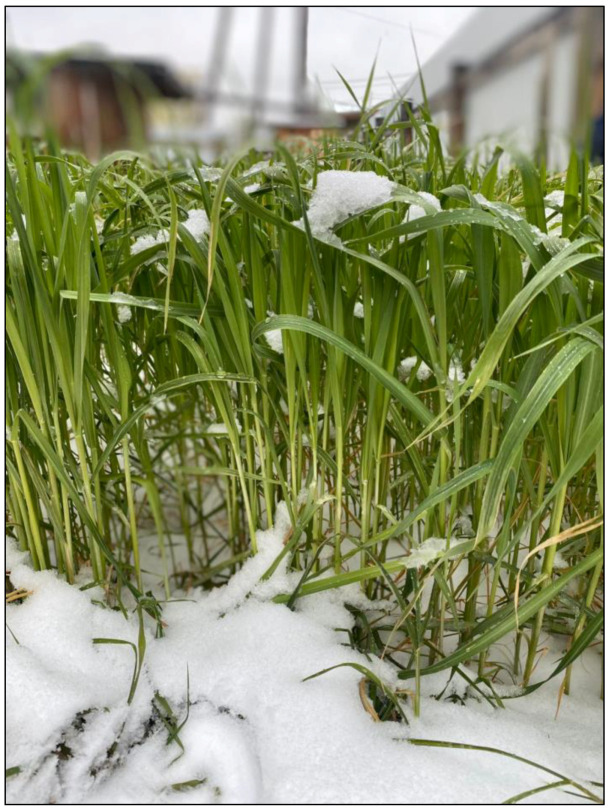
Late summer-sown *A. sativa* (Vilensky variety) under conditions of cryolithozone of Yakutia (Central Yakutia, Republic of Sakha (Yakutia), Russia).

As a result of field observations of phenological phases of development of late-sown oats (*A. sativa*), which in cryolithozone conditions were seeded in the middle of July (July 21), it was revealed that hardening by low positive temperatures (from 5.6 to 1.2 °C) took place in the middle and at the end of September (Figure 1), when the plants were in the tube emergence phase. When ambient temperatures subsequently decreased (from −0.1 to −5.1 °C) in October and snow cover was established, oat plants went under snow in the green state (Figure 2) during the tube emergence phase. During this period, in the conditions of the cryolithozone of Yakutia, there was not only a decrease in air temperature (from −0.1 to −5.1 °C), but also a reduction in the photoperiod and precipitation in the form of rain and snow. During the entire observation period, an average of 11.4 mm of precipitation in the form of rain and snow fell.

### 2.2. Metabolomic Changes in Oat Leaves during the Autumn Period with the Onset of Low Quenching Temperatures

**Figure 3:**
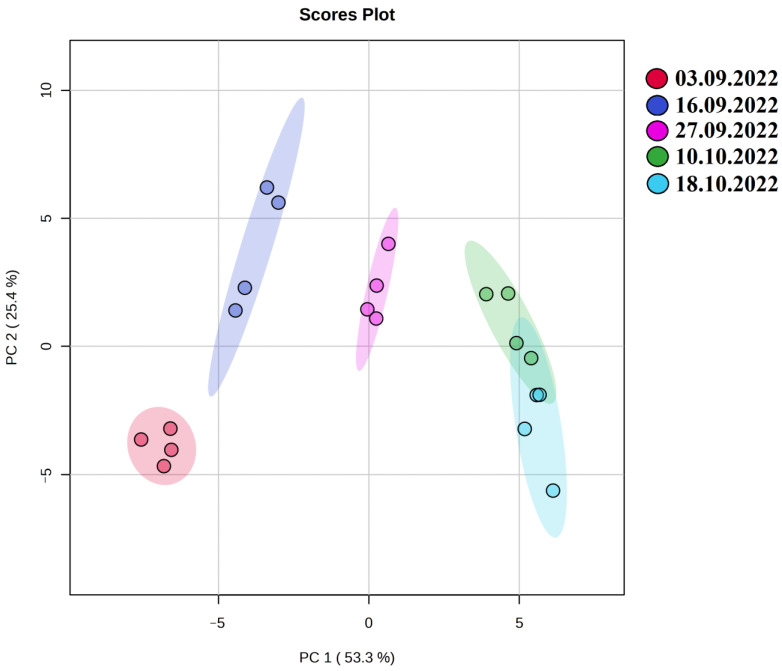
PCA score plot from principal component analysis of metabolite profiles of *А. sativa* in various phenological phases.

**Figure 4:**
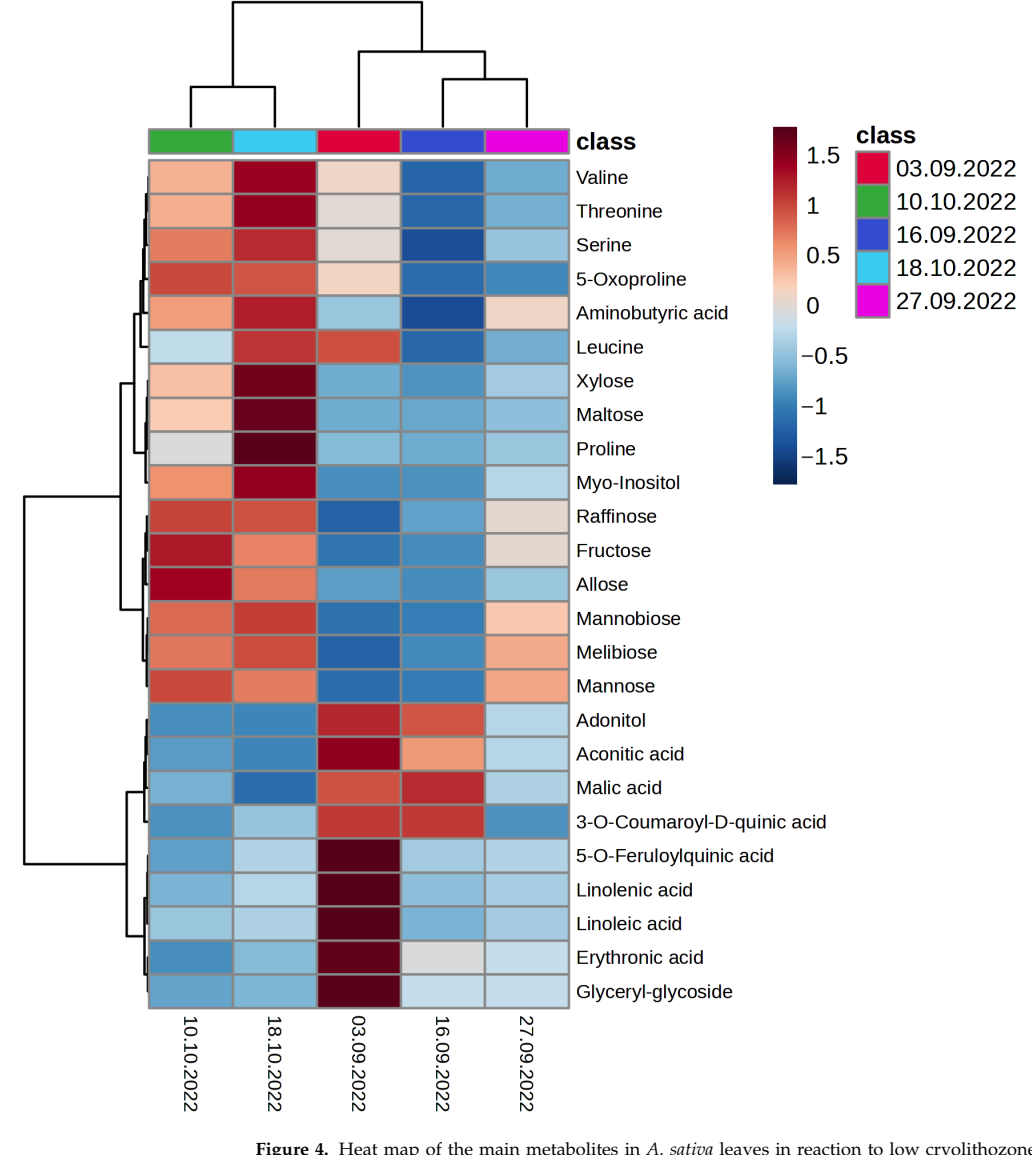
Heat map of the main metabolites in *A. sativa* leaves in reaction to low cryolithozone temperatures.

Metabolomic analysis of *A. sativa* leaf samples was performed using GC-MS. For statistical analysis of the obtained metabolomic data, a matrix including 20 observations for 41 metabolites was created (Table S1). The obtained data set was processed by the principal component analysis (PCA) method (Figure 3). It is shown that the obtained metabolomic profiles of *A. sativa* clustered as the ambient temperature decreased in September and October. It should be noted that the reflections of metabolomes of *A. sativa* on the graph of accounts selected on 10.10 and 18.10 overlapped with each other, which may indicate significant metabolic rearrangements, contributing to the hardening of the organism at low positive temperatures from 5.8 to 1.2 °C (3.09 to 27.09) and its slowing down with the onset of negative temperatures from −0.1 to −5.1 °C (10.10 to 18.10). As a result of metabolomic profiling of *A. sativa* leaves using GC-MS, 41 metabolites were identified (Figure 4; Table S1).

#### 2.2.1. Organic and Inorganic Acids

Among organic and inorganic acids, seven metabolites with different dynamics were detected in oat leaves. The content of phosphoric acid significantly decreased by 1.7 times at the onset of negative temperatures (−5.1 °C) in the middle of October compared to the beginning of September (5.8 °C). The amount of succinic acid decreased by 1.6 times (27.09) in comparison with the beginning of September (3.09), when near-zero ambient temperatures were established in the cryolithozone at the end of September (1.2 °C) (Figure 1). The amounts of other identified organic and inorganic acids, such as malic, aconitic, glyceric and itaconic acids, gradually decreased with the onset of fall cold weather (from −0.1 to −5.1 °C). On the contrary, the content of ascorbic acid increased by 8.5 times in late October compared to early September, at which time the average daily air temperature was −5.1 °C. In general, the composition of organic and inorganic acids in oat leaves was dominated by aconitic acid compared to other metabolites, the maximum content of which occurred in early September (5.8 °C) and was about 10 mg/g DW.

#### 2.2.2. Amino Acids

Ten metabolites were identified in the composition of amino acids in oat leaves. As a result of the studies, it was revealed that in response to low-temperature stress caused by a decrease in the average daily air temperature in September (from 5.8 to 1.2 °C) and October (from −0.1 to −5.1 °C) and a reduction in the photoperiod, the amounts of amino acids such as alanine and leucine significantly decreased in the studied oat plants. The amounts of valine and serine were low in mid-September, then their levels increased in October when negative ambient temperatures occurred. Other amino acids—proline, 5-Oxoproline and γ-Aminobutyric acid—showed the same tendency. 

#### 2.2.3. Fatty Acids and Lipid Residues

Five fatty acids (FAs) and one lipid residue (Glyceryl-glycoside) in late-sown oat leaves were identified as part of the metabolomic profile. During the observation period, the contents of palmitic (C16:0), linoleic (C18:2n − 6) and linolenic acids (C18:3n − 3) and glyceryl-glycoside in oat leaves decreased significantly with the gradual cooling of fall temperatures in mid-September and October (from 1.2 to −5.1 °C). The content of stearic acid (C18:0) did not change and remained practically at a constant level during the research period.

#### 2.2.4. Polyols 

In oat leaves, the polyols erythronic acid and adonitol tended to decrease in mid-September and in October. The ribitol content did not change in response to the natural decrease in ambient temperature in the Yakutia cryolithozone. Among polyols, the absolute content of myo-inositol increased in response to the fall weather cooling in mid-September and October (from 1.2 to −5.1 °C) compared to early September (5.8 °C).

#### 2.2.5. Monosaccharides 

The quantitative contents of all identified monosaccharides increased in response to the temperature decrease to near-zero degrees (27.09 (1.2 °C); 10.10 (−0.1 °C)) and sustained negative temperatures −5.1 °C (18.10). In oat leaves, fructofuranose, followed by mannopyranose and glucopyranose, prevailed among monosaccharides. Green leaves of late summer-sown oats contained xylose and allofuranose; the contents of these sugars were low compared to those of other identified monosaccharides and did not exceed 4.8 ± 0.5 mg/g DW in oat leaves.

#### 2.2.6. Disaccharides

In *A. sativa* leaves, the contents of disaccharides increased with the gradual decrease in temperature (from 5.6 to −5.1 °C), except for raffinose. The absolute sucrose contents of leaves sampled on 27 September were decreased by 1.3 times compared to leaves sampled on 3 September. Oat plants sampled on 27 September (1.2 °C) were stressed by the decrease in the average daily air temperature to near zero. Among disaccharides, sucrose contents were much higher than those of all other carbohydrates. Maltose showed the most noticeable increase with a gradual decrease in temperature among disaccharides and had increased by 9.3 times on 18 October (−5.1 °C) compared to the previous month (16 September) (5.6 °C).

#### 2.2.7. Phenolic Compounds

Using GC-MS to analyze late-sown oat leaves, five phenolic compounds were identified. Among the identified phenolic compounds in flag oat leaves, quinic acid was dominant; the content of this compound significantly increased in October by 1.6 times from October 10 to October 18 (during which period the temperature decreased from −0.1 to −5.1 °C) compared to the beginning of the experimental observations (3 September, when the temperature was 5.8 °C). In October, stable snow cover and low negative air temperatures were established in permafrost ecosystems. The content of 3-O-Feruloylquinic acid did not change significantly during the studied period. The amounts of compounds such as 3-O-Coumaroyl-D-quinic acid, 5-O-Feruloylquinic acid and chlorogenic acid decreased in response to negative air temperatures in October (from −0.1 to −5.1 °C).

#### 2.2.8. Correlation of the Contents of Metabolites Involved in Raffinose Synthesis with Mean Air Temperature

**Figure 5:**
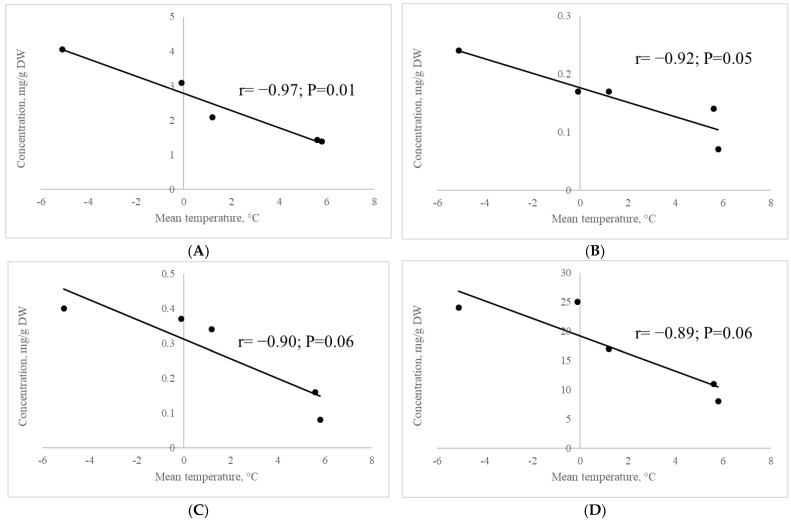
Correlation of myo-inositol (**A**), galactinol (**B**), melibiose (**C**) and raffinose (**D**) contents in *A. sativa* leaves with mean air temperature.

Inverse correlations of mean air temperature with the concentration of myo-inositol (r = −0.97; *p* = 0.01), galactinol (r = −0.92; *p* = 0.05), melibiose (r = −0.90; *p* = 0.06) and raffinose (r = −0.89; *p* < 0.06) in leaves of late summer-sown *A. sativa* oats growing in the cryolithozone area were detected (Figure 5).

### 2.3. Chlorophyll a Fluorescence Parameters (JIP Test)

**Figure 6:**
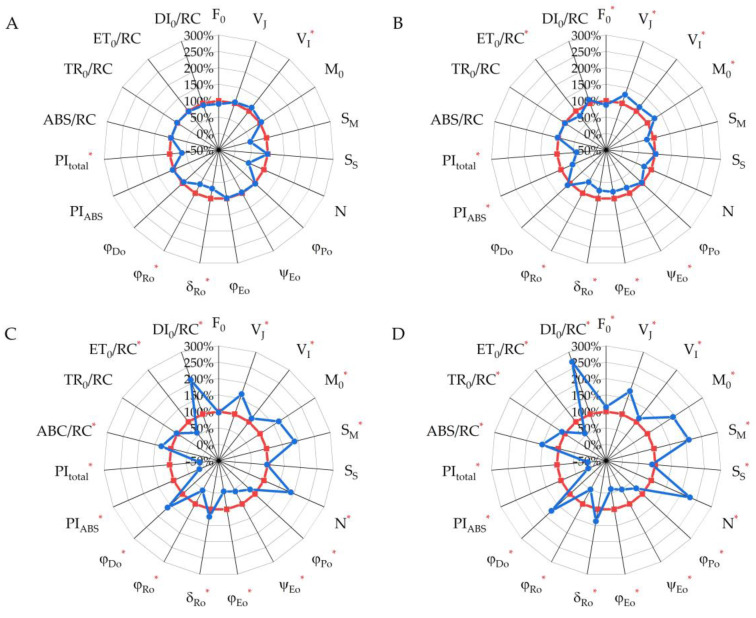
Spider plot of JIP-test parameters computed from chlorophyll *a* fluorescence of *A. sativa*. The red circle in the middle of each plot is at 3.9 and the blue lines are at 16.9 (**A**), 27.9 (**B**), 10.10 (**C**) and 18.10 (**D**). For each parameter, the value for 3.9 is set as 100%. Asterisks indicate significance of differences at *p* < 0.05 by Tukey’s multiple comparison test (Tukey’s HSD).

The OJIP curves obtained for chlorophyll *a* fluorescence induction were analyzed using the standard JIP-test protocol (Table 1). Analysis of the results showed that in early fall (3 and 16 September) (Figure 6A), there was an increase in parameter V_I_, which indicated an increase in the O-I phase of the OJIP curve of chlorophyll *a* fluorescence of oat leaves. In addition, the efficiency and quantum yield of electron transfer between PS II and PS I, the parameters δ_Ro_ and φ_Ro_, respectively, decreased, as a result of which the total performance index (PI_total_) decreased, among other indices. Comparative analysis between 3 and 27 September (Figure 6B) showed that there was a decrease in the initial fluorescence intensity (F_0_), in addition to the suppression of electron transfer between the photosystems (V_I_, δ_Ro_ and φ_Ro_); the parameter V_J_ increased, which was related to the efficiency and quantum yield of electron transport downstream of the electron acceptor (QA) on the acceptor side of PS II (ψ_Eo_, φ_Eo_ and PI_ABS_). The decrease in the efficiency of electron transport (ET) affected the increase in the initial slope of the OJIP curve (M_0_). The second decade of October (10 to 18 October) (Figure 6C,D) was characterized by a significant inactivation of ET within PS II and between photosystems (S_M_, ψ_Eo_, φ_Eo_, δ_Ro_ and φ_Ro_); a strong increase in non-photochemical quenching (DI_0_/RC and φ_Do_); and a strong decrease in performance indices (PI_ABS_ and PI_total_). It should be noted that there was a slight change in the trapped energy flux (TR_0_/RC) as well as in the parameter of maximum quantum yield for primary photochemistry (φ_Po_) in the studied leaves of oat plants.

### 2.4. Leaf Reflectance and Reflectance Indices

**Figure 7:**
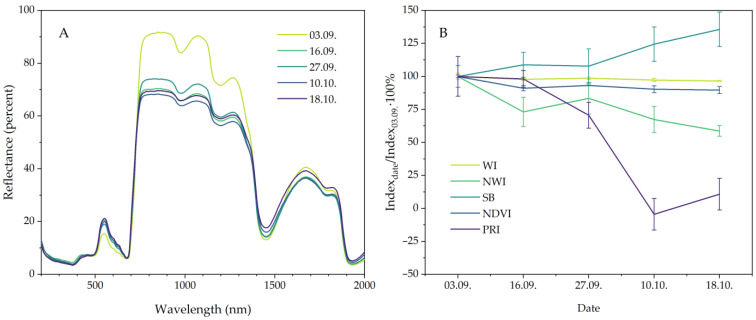
Autumn changes in reflectance spectra (**A**) and normalized leaf reflectance indices (**B**) of late summer-sown oats (*A. sativa*).

The reflectance indices most commonly used by other authors to characterize leaf condition (Table 1), such as the WI, NWI, single-band (SB), NDVI and PRI indices, were considered (Figure 7B). The water index (WI) relies on the absorption band in the 970 nm region, which is associated with changes in leaf water content, as does its normalized counterpart (NWI). Other authors often use different variations of the NDVI and PRI indices to characterize chlorophyll content, overall photosynthetic activity and biomass accumulation; these indices rely on wavelengths of 640 nm and 530 nm, respectively. The parameters reflecting direct water absorption (SB) are often determined using a wavelength of 1450 nm, which is associated with water absorption in plant tissues.

Comparing the results for the fall period showed a decrease in the NWI index and an increase in the SB index, while the WI index decreased slightly. The NDVI index remained almost unchanged during the fall period, while the PRI index decreased significantly in early October. Notably, at minimum temperatures below zero, the albedo of leaf reflectance in the NIR emission region decreased sharply as early as mid-September [26] (Figure 7A). This decrease is attributed to the general effect of deep cold stress on the plants, which apparently destroys the semi-permeability of cell membranes, while intercellular sap migrates into the intercellular space, reducing leaf reflectance of NIR radiation. Variation in the broad reflectance region also affects the NWI and SB indices at 1450 nm, which are correlated with water absorption bands [24].

## 3. Discussion

During adaptation to low temperatures (low positive and negative temperatures) in crops, biochemical reactions are associated with changes in metabolic pathways, which lead to the formation of sugars, the main energy substrates of respiration in plants. The increase in the content of sugars contributes to the retention of water in the cell in the unfrozen state, which prevents denaturation of proteins [27]. The contents of sugar alcohols (e.g., mannitol), amino acids (e.g., proline) and amines (e.g., glycine, betaine and polyamines) also increase with hypothermia [28]. These metabolites perform important physiological roles in the plant cell: (1) they are osmolytes, reducing cell dehydration; (2) they stabilize enzymatic reactions, cell membranes and other cellular components; and (3) they are chelating agents that bind metals and inorganic ions [29]. All these metabolic changes at low temperatures reflect changes in the functional state of leaves in PS II and in the whole electron-transport chain during photosynthesis [30].

A number of studies on different cereal species have shown that late sowing in wheat enhances lodging resistance by improving lignin and cellulose biosynthesis and accumulation [31] under Central European (Poland) conditions. Late sowing of spring oats (1 September) gave the best forage value but the lowest dry matter yield for the three different varieties studied; in addition, our previous studies showed that late-sown *A. sativa* (sown 15 July under cryolithozone conditions) is the most suitable species among other cereals for obtaining lutein concentrate from leaves frozen by natural cold in the cryolithozone [32]. 

In the autumn period, the reduction in the photoperiod (light levels), along with low temperatures, are stress factors for leaves of herbaceous plants growing in the cryolithozone, which lead to significant changes in the metabolism of the plant organism as a whole. Amino acids are known to be actively involved in plant adaptive responses to various stresses and environmental signals (light, biotic and abiotic stresses) [33]. Thus, it has been found that an increased Proline content in plant cells can be used as an indicator of metabolic processes [33], and its ability to accumulate has been shown to correlate with abiotic stress tolerance [34]. In experiments with oat leaves, these results were confirmed. Proline content correlated with decreasing air temperature.

During the action of low positive ambient temperatures on plants, the process of formation of cryoprotectors in cells is most pronounced. Their role in plant cells is usually performed by low- and high-molecular compounds: carbohydrates, lipids, proteins and other compounds. Due to cryoprotectors, the cell becomes more protected from intracellular ice formation and dehydration. At the same time, the content of carbohydrates, the main energy substrates of respiration in plants, increases especially intensively. The increase in carbohydrate content contributes to the retention of water in the cell in the unfrozen state, which prevents denaturation of proteins [35].

It is known that some polyols can exhibit cryoprotective functions in plants, e.g., methylmucoinositol contents in *Viscum album* leaves increased when exposed to low-temperature stress [36]. Orthen and Popp [37] showed that some polyols are effective in protecting the thylakoid membrane when exposed to low temperatures.

The adaptive capacity of plants is highly dependent on their ability to maintain membrane fluidity and prevent lipid phase transition under stressors [38]. The disruption of cell membrane integrity and the subsequent leakage of intracellular contents can have dramatic consequences for the cell and the organism as a whole. The structural rearrangements in cell membranes under the influence of low temperatures significantly affect fatty acid lipids [39]. In the present experiments, a significant decrease in FAs, such as C16:0, C18:2n−6 and C18:3n−3, with the onset of fall quenching cold weather and a decrease in leaf reflectance in the NIR emission region were detected. These changes indirectly indicate the disruption of cell membrane integrity.

Inhibition of light-induced photosynthetic processes occurred sequentially; first of all, cold stress reduced the efficiency of electron transport between PS II and PS I. This may have been due to leakage of intracellular contents, which affects the efficiency of this process. Later, with the onset of cold weather, the efficiency of the acceptor side of PS II and then the donor side of PS II decreased as a result of photoinhibition and photooxidative stress. In previous studies, cold stress was previously shown to inhibit linear electron transport, whereas cyclic electron transport around PS I was enhanced, which contributes to the adaptive biochemical changes that occur as a result of walking stress [40]. The general change in the photosynthetic activity of leaves and the accumulation of protector substances (sugars) in oat leaves are in good agreement with the decrease in the reflection index (PRI), which in turn can serve as a good indicator of possible rearrangements in the photochemical processes and biochemical composition of leaves.

The method of principal components established a distinct separation of the metabolomes of *A. sativa* collected on 10 and 18 October, when the average air temperature was below 0 °C. The distinct separation of *A. sativa* metabolomes at low positive temperatures (3.09 to 29.09) indicated metabolic rearrangements that contribute to hardening of the organism before the onset of negative temperatures. It was shown that metabolic processes in *A. sativa* tissues are slowed down but proceed at negative temperatures as well. A thermomap (Figure 4) shows that at negative ambient temperatures, mono- and oligosaccharides, polyols and amino acids accumulate in *A. sativa* cells, which most likely contribute to plant resistance to low-temperature stress.

Fischer and Höll [41] showed that the accumulation of melibiose and raffinose in the pine needles of *Pinus sylvestris* L. was observed only during winter, which may indicate their cryoprotective function in the plant body. Raffinose family oligosaccharides are known to contribute to membrane stability under low temperature stress by insertion between lipid headgroups [42]. Even small concentrations of raffinose have been found to reduce the rate of crystallization of sugars [43], and thus synthesis of oligosaccharides of the raffinose family may contribute to plant resistance to low temperatures. Average air temperature was found to be inversely correlated with the concentration of myo-inositol (r = −0.97; *p* = 0.01), galactinol (r = −0.92; *p* = 0.05), melibiose (r = −0.90; *p* = 0.06) and raffinose (r = −0.89; *p* < 0.06) in *A. sativa* leaves in the cryolithozone area (Figure 5). It is known that myo-inositol and galactinol are substrates for the synthesis of raffinose and melibiose in plant tissues, so their concentration also increases with decreasing air temperature. Thus, it has been shown that the synthesis of oligosaccharides of the raffinose family in *A. sativa* tissues in the cryolithozone area, including melibiose and raffinose, is activated by low ambient temperatures, which contributes to cryoprotection of the organism.

## 4. Materials and Methods

### 4.1. Oat Variety Characteristics

Currently, out of 30 spring oat varieties included in the State Register of Selection Achievements in the East Siberian region, 3 varieties are cultivated in Yakutia: Pokrovsky, Pokrovsky 9 and Vilensky. The Vilensky oat variety was selected for these experiments. This variety was created at the Yakutsk Research Institute of Agriculture by hybridization of the locally released variety Pokrovsky 9 × (Wodan × Khibiny 2). The creators of the variety are Petrova L.V., Rozhin V.S. and Danilova V.P. It is included in the State Register for the East Siberian (XI) region. It is distributed in all agricultural zones of the Republic of Sakha (Yakutia). It has a 1000-grain weight of 31–38 g. It is medium-early, with a vegetation period 65–70 days. The resistance to lodging is at the level of the standard variety, Pokrovsky. The protein content is 10.2–11.9%, and the grain weight is 460–580 g/L. It is moderately susceptible to dusty and durum bunt. Under field conditions, it was poorly affected by bacterial blight, red-brown spot and stem rust. The average yield in the East Siberian region was 21.6 c/ha. In the Republic of Sakha (Yakutia), the gain to the standard Pokrovsky amounted to 2.5 c/ha, with a yield of 25.7 c/ha. The maximum yield (61.2 c/ha) was obtained in 2014 in the Transbaikal Territory.

### 4.2. Cultivation Conditions and Field Experiment

Research location: Russia, Central Yakutia, Yakutsk, suburban area (62° N and 130° E). The average air temperature for the growing season (June–September) was 14.1 °C, and the sum of precipitation for May–September was 122.5 mm. In May, the amount of precipitation was 10.3 mm. In June, July, August and September, 19.9, 20.2, 51 and 21.1 mm of precipitation occurred, respectively. 

*A. sativa* plants were grown on an experimental plot (plot area: 6 m^2^) located on the middle floodplain of the Lena River without irrigation (Figure 1). The site soils were floodplain meadow-black earth soils formed on light loam. Agrotechnics for the cultivation of late summer-sown oats were carried out according to the agrotechnological scheme generally accepted in Central Yakutia [44]. The seeds were sown on a late date (21 July). Plants of this sowing date were in the tillering phase at the onset of freezing temperatures in early October. Frozen green flag leaves of oats that underwent cold hardening (+8 to −5 °C) from early September to the first days of October were used for biochemical analyses. Exposure to temperatures of −5–−10 °C resulted in freezing and death of plants within a day (8–24 h).

### 4.3. Sample Collection and Storage Prior to Analysis 

The samples were collected in the first half of the day (9:00–11:00). The samples were immediately fixed in liquid nitrogen and transported in Dewar vessels to the laboratory. 

The samples were stored in a freezer at −86 °C (BioBase, China) until analysis. For biochemical studies, the samples of *A. sativa* leaves fixed in liquid nitrogen were dried in the lyophilizer (BioBase, China). The experiments were carried out in 2022. The data on air temperatures in the habitats of herbaceous plants were taken from an Internet resource (http://www.pogodaiklimat.ru/weather.php?id=24959 (accessed on 8 February 2024)). Weather conditions in the years of the experiment were typical for Central Yakutia (Table 1).

### 4.4. Metabolomic Analysis of Leaves of Late Summer-Sown A. sativa

To perform a metabolomic analysis, 10 mg of freeze-dried leaves of *A. sativa* was extracted in 1 mL of methanol. The extract was evaporated at 40 °C on a rotary evaporator. The dry residue was dissolved in 50 μL of pyridine in a vial. Then, trimethylsilyl (TMS) derivatization was performed by adding 50 μL of N,O-bis(trimethylsilyl)trifluoroacetamide (BSTFA) into the vial. The vial was then heated at 100 °C for 15 min. Mass spectra were obtained using gas chromatography mass spectrometry (GC-MS) on an Agilent 7820/5975 GC-MS system (Agilent Technologies, Santa Clara, CA, USA), which was equipped with an HP-5MS column (30 m × 0.25 mm × 0.25 μm). The injector temperature was set at 250 °C. A linear temperature gradient was established for column heating, increasing from 70 °C to 320 °C with a heating rate of 4 °C/min. Helium was used as a carrier gas and had a constant flow of 1 mL/min. Data collection was performed using Agilent ChemStation E.02.02.1431 software (Agilent Technologies, Santa Clara, CA, USA). The quantitative interpretation of the chromatograms was carried out using the internal standardization method for hydrocarbon C23 [45]. The processing and interpretation of the mass spectrometric information was carried out using the NIST 2017 standard library. Statistical processing of metabolomic profiles was performed using the principal component method in the MetaboAnalyst resource (www.metaboanalyst.ca (accessed on 8 March 2024)).

### 4.5. Measuring Chlorophyll a Fluorescence Parameters in Leaves

Induction curves of chlorophyll *a* fluorescence of sown oat leaves were measured on a portable fluorimeter-FluorPen FP 100 (Photon Systems Instruments, Brno, Czech Republic). Measurements were performed after 15 min of dark adaptation of the samples, and at least 20 repetitions were measured for each date. A saturating flash was used with an intensity of 3000 μmol photons m^−2^ s^−1^, the excitation wavelength λ = 455 nm, and the irradiation measurement had a duration of 1 s. To analyze the OJIP curve of chlorophyll *a* fluorescence, the following parameters of the JIP test were used, as shown in Table 1.

### 4.6. Measuring Leaf Reflectance and Computing Reflectance Indices

Leaf reflectance was measured on a Lambda 750S spectrophotometer with an integrating sphere (Perkin Elmer Inc., Waltham, MA, USA). The measurement range was from 200 to 2000 nm, with a spectral resolution of 5 nm and 360 spectral bands. The oat plants were transported to the laboratory together with soil inside a thermostatted container, and measurements were carried out within 1 h from the moment of plant removal from the experimental plot. Reflectance measurements of oat leaves for each date were made at least three times, with averaging from 3–4 leaves. The reflectance indices were calculated according to the following formulas presented in Table 1. 

### 4.7. Data Analysis

The obtained data were processed by one-factor analysis of variance with a significance level of 0.05 in Microsoft Excel 2010. Figures were plotted using the OriginPro 2021 software package (OriginLab, Northampton, MA, USA) and Microsoft Excel 2010. Figures represent arithmetic mean values and their standard deviations. All calculations comparing JIP-test parameters were performed in STATISTICA version 10.0 software (TIBCO Software Inc., Palo Alto, CA, USA). Biochemical parameters were determined in 3 biological samples (4 analytical replicates each).

## 5. Conclusions

**Figure 8:**
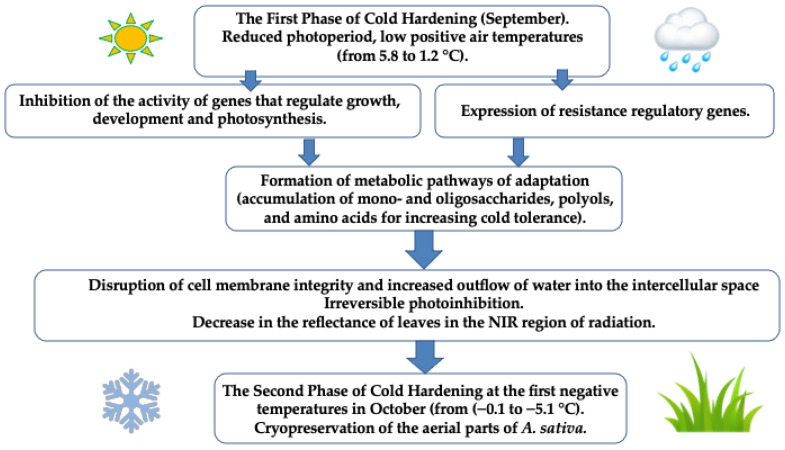
General scheme of cold hardening of *A. sativa* occurring during adaptation to low temperatures of the permafrost zone.

Metabolomic profiling serves as a metabolic fingerprint of physiological and biochemical events occurring in the plant organism in response to stressful environmental conditions. Cold hardening of *A. sativa* in permafrost ecosystems was limited to a short fall period of about 4 weeks. It was revealed that oat plants during this period gradually reprogramed their metabolism as the ambient temperature decreased (Figure 4). Oat plants stressed by near-zero and subzero temperatures showed the most pronounced accumulation of mono- and oligosaccharides, polyols and amino acids to increase cold tolerance. During this period, there was also a significant decrease in the sum of FAs, such as C16:0, C18:2n−6 and C18:3n−3, as well as a decrease in the reflectance of leaves in the NIR region of radiation. It is believed that these changes indicate the disruption of cell membrane integrity and increased outflow of water into the intercellular space. Simultaneously, the efficiency of transport from intersystem electron transporters to the acceptor side of PS I decreased, and later a sequential decrease in the activity of all linear electron transport was observed. Thus, one of the peculiarities of cold acclimation of late summer cereals growing in cryolithozone conditions is obviously the high accumulation of primary and secondary metabolites (mono- and oligosaccharides, polyols and amino acids) in cells and general rearrangements in the photosynthetic activity of leaves (Figure 8). The experimental data obtained in this work can serve as good indicators (through the assessment of photochemical processes and metabolomic profiling) in the selection of valuable species and for obtaining plant forages with high contents of useful substances under conditions of a sharply continental climate. 

## Figures and Tables

**Table 1:** Formulae and explanations of selected JIP-test [13,14] and reflectance indices used in this research.

Parameters/Indices	Formula	Definition
JIP-test parameters		
F_0_	F_0_ = F_50µs_	Minimal fluorescence, when all PS II RCs are open (at t = 0)
V_J_	V_J_ = (F_J_ − F_0_)/(F_M_ −F_0_)	Relative variable fluorescence at 3 ms, called the J phase
V_I_	V_I_ = (F_I_ − F_0_)/(F_M_ − F_0_)	Relative variable fluorescence at 30 ms, called the I phase
M_0_	M_0_ ≡ 4(F_300µs_ − F_50µs_)/(F_M_ − F_0_)	Initial slope of the fluorescence intensity at 50–300 µs
S_M_	S_M_ = Area/(F_M_ − F_0_)	Normalized total area above the O-J-I-P fluorescence transient, reflects the capacity of the electron acceptor pool
S_S_	S_S_ = V_J_/M_0_	Single-turnover Q_A_ reduction events
N	N = S_M_/S_S_ = S_M_ M_0_ (1/V_J_)	Turnover number: number of Q_A_ reduction events between time 0 and $t_{F_{M}}$
φ_Po_	φ_Po_ ≡ TR_0_/ABS = [1 − (F_0_/F_M_)]	Maximum quantum yield shows the efficiency of the primary charge separation processes at PS II RCs
ψ_Eo_	ψ_Eo_ ≡ ET_0_/ABS = [1 − (F_0_/F_M_)](1 − V_J_)	Efficiency/probability that an electron $\mathrm{moves}\;\mathrm{further}\;\mathrm{than}\;Q_{A}^{-}$
φ_Eo_	φ_Eo_ = ET_0_/ABS = [1 − (F_0_/F_M_)] ψ_Eo_	Quantum yield of electron transfer beyond the acceptor Q_A_ of PS II (ET)
δ_Ro_	δ_Ro_ ≡ RE_0_/ET_0_ = (1 − V_I_)/(1 − V_J_)	Probability of electron transfer to the acceptor side of PS I(RE)
φ_Ro_	φ_Ro_ ≡ RE_0_/ABS = [1 − (F_0_/F_M_)] (1 − V_I_)	Quantum yield of electron transfer to the acceptor side of PS I(RE)
φ_Do_	φ_Do_ = 1 − φ_Po_	The fraction of dissipated energy of PS II
PI_ABS_	${\mathrm{PI}}_{\mathrm{ABS}}=\frac{\mathrm{RC}}{\mathrm{ABS}}\frac{\varphi_{\mathrm{Po}}}{1-\varphi_{\mathrm{Po}}}\frac{\psi_{\mathrm{Eo}}}{1-\psi_{\mathrm{Eo}}}$	Performance index reflects the conversion of energy photons absorbed by PS II to electron flow further from acceptor Q_A_
PI_total_	${\mathrm{PI}}_{\mathrm{total}}={\mathrm{PI}}_{\mathrm{ABS}}\frac{\delta_{\mathrm{Ro}}}{1-\delta_{\mathrm{Ro}}}$ _*_	Total performance index reflects the conversion of energy photons absorbed by PS II acceptor side of PS I
ABS/RC	ABS/RC = M_0_ (1/V_J_) (l/φ_Po_)	Absorption flux per active RC, reflects the relative size of the antenna
TR_0_/RC	TR_0_/RC = M_0_ (1/V_J_)	Trapped energy flux of photons absorbed by PS II per active RC
ET_0_/RC	ET_0_/RC = M_0_ (1/V_J_) ψ_Eo_	Electron transport flux further of acceptor Q_A_ per active RC
DI_0_/RC	DI_0_/RC = (ABS/RC) − (TR_0_/RC)	Dissipated energy flux per active RC
Reflectance indices		
Water index (WI)	WI = R_900_/R_970_	Index based on the water absorption band at 970 nm, reflecting the relative water content in plant leaves [15,16]
Normalized water index (NWI)	NWI = (R_970_ − R_900_)/(R_970_ + R_900_)	Normalized index based on the water absorption band at 970 nm, reflecting the relative water content in plant leaves [17,18,19]
Normalized difference vegetation index (NDVI)	NDVI = (R_860_ − R_640_)/(R_860_ + R_640_)	The index, which is an indicator of nitrogen content and biomass accumulation, also shows the water content in leaf tissues [20,21]
Photo-chemical reflectance index (PRI)	PRI = (R_530_ − R_570_)/(R_530_ + R_570_)	Index related to the influence of ecophysiological factors on the photosynthetic efficiency of plants [22,23]
Single band (SB)	SB = R_1450_/100	Single band at 1450 nm related to water absorption [24,25]

## Data Availability

All data are contained within the article.

## Supplementary Materials

The following supporting information can be downloaded at: https://www.mdpi.com/article/10.3390/plants13081076/s1.
