# Supplementary material for: Metabolomic Profile and Functional State of Oat Plants (Avena sativa L.) Sown under Low-Temperature Conditions in the Cryolithozone
Source: Plants (Basel). 2024 Apr 11;13(8):1076. doi: 10.3390/plants13081076 (PMC11054449; doi:10.3390/plants13081076)
Supplement: Supplementary file 1 [file plants-13-01076-s001.zip › plants-2964083-supplementary.pdf]

Table S1. Metabolites and their chemical formulas from *A. sativa* leaves

№	t_R, min	Compound	Chemical formula
1	7.01	Alanine	$C_3H_7NO_2$
2	9.37	Valine	$C_5H_{11}NO_2$
3	10.59	Leucine	$C_6H_{13}NO_2$
4	10.69	Phosphoric acid	H_3PO_4
5	11.06	Proline	$C_5H_9NO_2$
6	11.44	Succinic acid	$C_4H_6O_4$
7	11.95	Glyceric acid	$C_3H_6O_4$
8	12.09	Itaconic acid	$C_5H_6O_4$
9	12.57	Serine	$C_3H_7NO_3$
10	13.15	Threonine	$C_4H_9NO_3$
11	15.25	Malic acid	$C_4H_6O_5$
12	15.79	5-Oxoproline	$C_5H_7NO_3$
13	15.93	Aminobutyric acid	$C_4H_9NO_2$
14	16.45	Erythronic acid	C4H8O5
15	17.79	Glutamic acid	$C_5H_9NO_4$
16	19.30	Xylose	$C_5H_{10}O_5$
17	19.89	Ribitol	$C_5H_{12}O_5$
18	19.99	Adonitol	$C_5H_{12}O_5$
19	20.15	Aconitic acid	$C_6H_6O_6$
20	21.55	Fructose	$C_6H_{12}O_6$
21	22.11	Allose	$C_6H_{12}O_6$
22	22.34	Quininic acid	$C_{11}H_9NO_3$
23	22.88	Mannose	$C_6H_{12}O_6$
24	23.65	Ascorbic acid	$C_6H_8O_6$
25	24.38	Glucose	$C_6H_{12}O_6$
26	24.82	Palmitic Acid	$C_{16}H_{32}O_2$
27	25.99	Myo-Inositol	$C_6H_{12}O_6$
28	27.29	Linoleic acid	$C_{18}H_{32}O_2$
29	27.40	Linolenic acid	$C_{18}H_{30}O_2$
30	27.75	Stearic acid	$C_{18}H_{36}O_2$
31	29.41	Glyceryl-glycoside	$C_9H_{18}O_8$
32	32.02	Maltose	$C_{12}H_{22}O_{11}$
33	33.80	Sucrose	$C_{12}H_{22}O_{11}$
34	35.00	Mannobiose	$C_{12}H_{22}O_{11}$
35	36.34	Melibiose	$C_{12}H_{22}O_{11}$
36	37.42	3-O-Feruloylquinic acid	$C_{17}H_{20}O_9$
37	37.84	Galactinol	$C_{12}H_{22}O_{11}$
38	38.34	3-O-Coumaroyl-D-quinic acid	$C_{16}H_{18}O_8$
39	39.64	5-O-Feruloylquinic acid	$C_{17}H_{20}O_9$
40	39.82	Chlorogenic acid	$C_{16}H_{18}O_9$
41	42.22	Raffinose	$C_{18}H_{32}O_{16}$
